# Elevated body fat increases amphetamine accumulation in brain: evidence from genetic and diet-induced forms of adiposity

**DOI:** 10.1038/s41398-021-01547-9

**Published:** 2021-08-14

**Authors:** Xiuping Fu, Aparna P. Shah, Jacqueline Keighron, Ta-Chung M. Mou, Bruce Ladenheim, Jesse Alt, Daisuke Fukudome, Minae Niwa, Kellie L. Tamashiro, Gianluigi Tanda, Akira Sawa, Jean-Lud Cadet, Rana Rais, Jay M. Baraban

**Affiliations:** 1 Solomon H. Snyder Department of Neuroscience, Johns Hopkins School of Medicine, Baltimore, MD 21205 USA; 2 Medication Development Program, National Institute on Drug Abuse, Intramural Research Program, National Institutes of Health, 333 Cassell Drive, Baltimore, MD 21224 USA; 3 Molecular Neuropsychiatry Research Branch, Intramural Research Program, NIDA/NIH/DHHS, 251 Bayview Boulevard, Baltimore, MD 21224 USA; 4 John Hopkins Drug Discovery, Department of Neurology, Johns Hopkins School of Medicine, Baltimore, MD 21205 USA; 5 Department of Psychiatry and Behavioral Sciences, Johns Hopkins School of Medicine, Baltimore, MD 21205 USA; 6 Department of Psychiatry and Behavioral Neurobiology, University of Alabama at Birmingham, Birmingham, AL 35233 USA

**Keywords:** Psychiatric disorders, Molecular neuroscience

## Abstract

Despite the high prevalence of obesity, little is known about its potential impact on the pharmacokinetics of psychotropic drugs. In the course of investigating the role of the microRNA system on neuronal signaling, we found that mice lacking the translin/trax microRNA-degrading enzyme display an exaggerated locomotor response to amphetamine. As these mice display robust adiposity in the context of normal body weight, we checked whether this phenotype might reflect elevated brain levels of amphetamine. To assess this hypothesis, we compared plasma and brain amphetamine levels of wild type and *Tsn* KO mice. Furthermore, we checked the effect of diet-induced increases in adiposity on plasma and brain amphetamine levels in wild type mice. Brain amphetamine levels were higher in *Tsn* KO mice than in wild type littermates and correlated with adiposity. Analysis of the effect of diet-induced increases in adiposity in wild type mice on brain amphetamine levels also demonstrated that brain amphetamine levels correlate with adiposity. Increased adiposity displayed by *Tsn* KO mice or by wild type mice fed a high-fat diet correlates with elevated brain amphetamine levels. As amphetamine and its analogues are widely used to treat attention deficit disorder, which is associated with obesity, further studies are warranted to assess the impact of adiposity on amphetamine levels in these patients.

## Introduction

Recent studies have focused attention on the role of the microRNA system in the pathophysiology of psychiatric diseases [1–4]. However, our understanding of how microRNA signaling pathways operate normally in the nervous system is quite limited, making it difficult to assess how altered microRNA signaling may contribute to these disorders. To help elucidate the physiological function of microRNA signaling in the brain, we are investigating the role of the translin (TN)/trax (TX) microRNA-degrading enzyme, an RNase that targets a small population of microRNAs [5, 6]. We are focusing on this enzyme because the expression of TN and TX is enriched in the brain, where it is localized preferentially to neurons [7].

Characterization of *translin* (*Tsn*) knockout (KO) mice, which lack the TN/TX complex, has enhanced our understanding of the role of this microRNA-degrading enzyme in vivo. These studies have revealed several prominent phenotypes displayed by *Tsn* KO mice: blockade of several forms of long-lasting synaptic plasticity [8, 9], decreased anxiety [10], protection from vascular stiffening [11], and development of robust adiposity in the context of normal body weight [12]. Furthermore, defining the mechanism mediating these phenotypes suggests that TN/TX operates in the following manner: TN/TX activation by cellular stimulation triggers rapid degradation of a small population of microRNAs. Degradation of these microRNAs reverses silencing of their target mRNAs, allowing *de novo* protein translation to proceed. Thus, the TN/TX complex appears to be well-positioned to mediate translation-dependent forms of cellular plasticity.

In pilot studies, we noted that TN and TX are expressed in midbrain dopaminergic (DA) neurons and striatal neurons, among other brain regions. These neuronal populations play key roles in mediating the action of psychostimulants [13–15]. Furthermore, recent studies have yielded compelling evidence demonstrating that the microRNA system plays a prominent role in mediating behavioral responses to these drugs [16–20]. Accordingly, we proceeded to check the locomotor response to amphetamine in *Tsn* KO mice. Although we found that constitutive *Tsn* KO mice display an exaggerated locomotor response to amphetamine, conditional deletion of *Tsn* in adulthood does not, suggesting that the amphetamine phenotype is due to a developmental effect of *Tsn* deletion. As we had reported previously that conditional deletion of *Tsn* in adulthood does not phenocopy the robust adiposity induced by constitutive *Tsn* deletion, we investigated whether the exaggerated response to amphetamine might be secondary to elevated adiposity.

## Methods and materials

### Animals

All mice were housed in ventilated racks and maintained on a 14 h/10 h light/dark cycle with access to regular chow (2018SX Teklad Global, Frederick, MD) and water *ad libitum*. All procedures were performed in accordance with the NIH Guide for the Care and Use of Laboratory Animals and approved by the Johns Hopkins Animal Care and Use Committee. Both male and female mice were used for open-field locomotor assays, as indicated. Only male mice were used in all other assays.

The *Tsn* KO mice used in these studies are from the line generated in Dr. Kasai’s laboratory [21] and provided by the JCRB Laboratory Animal Resource Bank of the National Institute of Biomedical Innovation (translin KO: Nbio055).

Mice with floxed alleles of *Tsn* or *Tsnax* were generated on a C57BL/6 J background using CRISPR/Cas9 technology as described in Fu et al. [22]. The following Cre driver or reporter lines were obtained from JAX labs or MMRRC (Drd1-Cre (EY217); Ai9 #007909; DAT-Cre #006660; Adora2a-Cre #036158; Ubiquitin C-CreERT2 #007001). Genotyping was performed on tail snips by Transnetyx, Inc. (Cordova, TN).

To examine the effects of elevated adiposity in WT mice (C57BL6/J), we separated 3-month-old mice obtained from JAX labs into three groups as described by Fordahl et al. [23]: regular chow, unrestricted access to high-fat (HF) diet (D12492; Research Diets, New Brunswick, NJ), and limited access to the same HF diet. The limited access mice had their feed changed from regular chow pellets to the HF pellets for a 2 h period on Monday, Wednesday and Friday. Mice were kept on this regimen for 7 weeks. All mice underwent scanning to determine their adiposity (% body fat) 5 days after being switched back to regular chow. After being scanned for body composition, mice underwent either open field locomotor testing or a collection of plasma and brain samples that were used for the determination of amphetamine levels.

### Immunostaining

Three to four-month-old male mice were deeply anesthetized with chloral hydrate (400 mg/kg, i.p.) for transcardial perfusion with 4% paraformaldehyde in 0.1 M phosphate-buffered saline pH 7.4 (PBS). Their brains were dissected and stored in 4% paraformaldehyde for at least 24 h, and then washed with PBS and transferred to 30% sucrose in PBS. After the brains totally sank, 30 μm sections were cut with a freezing microtome.

For TN immunostaining, sections underwent heat-induced antigen retrieval by incubating them in 10 mM NaCitrate for 30 min at a temperature of 70 °C. After the sections cooled down to room temperature, they were washed with PBS three times, blocked with 3% BSA and 0.1% Triton X-100 in PBS for 1 h, and then incubated with primary antibodies against TN [24] and tyrosine hydroxylase (TH; MilliporeSigma, Burlington, MA) overnight at 4 °C. After three washes, the sections were incubated with Alexa Fluor 488 goat anti-rabbit (Jackson ImmunoResearch Laboratories, West Grove, PA) and Alexa Fluor 594 goat anti-mouse (Jackson ImmunoResearch Laboratories) in 1.5% Normal Goat Serum (Vector Laboratories, Burlingame, CA) in PBS for 1 h at room temperature. After three additional washes with PBS, the sections were mounted on slides, and coverslipped.

For TX immunostaining, sections were washed with PBS three times and blocked with 3% BSA and 0.1% Triton X-100 in PBS for 1 h, followed by incubation with primary antibodies against TX [25] overnight at 4 °C. After three washes, the sections were incubated with biotinylated goat anti-rabbit (Vector Laboratories) in PBS for 1 h at room temperature. After three washes with PBS, the sections were incubated with AB solution (ABC kit, Vector Laboratories) for 90 min. Sections were washed with TBS three times and incubated with tyramide (TSA kit, PerkinElmer) for 10 min. After one wash with TBS and three washes with PBS, sections were mounted on slides, and coverslipped.

All images were obtained with a Zeiss LSM 800 confocal microscope.

### Western blotting

Forebrain, nucleus accumbens, and dorsal striatum tissue samples were homogenized in RIPA buffer (Cell Signaling Technology, Danvers, MA) containing a cocktail of protease and phosphatase inhibitors (MilliporeSigma). The concentration of total protein was determined using the Pierce BCA Protein Assay Kit (Thermo Fisher Scientific, Waltham, MA). Equal amounts of total protein were separated electrophoretically on an SDS-PAGE gel, transferred to a PVDF membrane (Bio-Rad, Hercules, CA) and immunoblotted with antibodies: anti-DAT (MilliporeSigma), anti-D2R (MilliporeSigma), anti-TH (MilliporeSigma), anti-actin (MilliporeSigma), anti-GAPDH (MilliporeSigma), and anti-TN. Blots were developed with the ECL system (Thermo Fisher Scientific). Band intensities were quantified from digital images by densitometry using Image J.

### Open-field locomotor activity

Changes in locomotor activity in response to amphetamine were assessed in an open field arena (50 × 50 cm). A 3 to 4-month old male or female mice were first allowed to habituate to the arena for 30 min. Following habituation to the context, mice were given an i.p. injection of saline and then placed back in the center of the open field and allowed to explore the arena for another 30 min. At this point, mice were given an intraperitoneal (i.p.) injection of D-amphetamine hemisulfate (2.5 mg/kg of total body weight or 3.5 mg/kg of lean mass; MilliporeSigma), placed back in the arena and then monitored for an additional 30–50 min.

### Measurement of tissue dopamine levels

Mice were sacrificed, their brains removed and placed on ice. Dorsal striatum and nucleus accumbens were dissected and weighed, then flash-frozen and stored at −80 °C. Tissue samples were ultrasonicated in 0.1 M perchloric acid and stored at −80 °C until extraction. Upon thawing, the samples were homogenized in 0.1 M perchloric acid and centrifuged at 25,000 g for 12 min. Dopamine levels were measured by HPLC with electrochemical detection. The analytical column was a SunFire C18 5 lm (4.6–150.0 mm) from Waters (Milford, MA, USA). The mobile phase was 0.01 M sodium dihydrogen phosphate, 0.01 M citric acid, 1.2 mM sodium EDTA, 1.2 mM sodium 1- heptane sulfonic acid, 10% methanol, pH 3.5; the flow rate was 1.0 mL/min and the column temperature was 34 °C. The installation consisted of a Waters 717 Plus automated injection system, a Waters 1525 Binary pump, and an ESA Coulochem III detector (Dionex, Sunnyvale, CA, USA). Waters Breeze system was used for analysis.

### Measurement of plasma and brain amphetamine levels

Amphetamine levels in plasma and brain samples were measured using high-performance liquid chromatography with tandem mass spectrometry (LC/MS-MS). Methanol, containing 0.5 µM losartan as an internal standard, was used to extract amphetamine from plasma and brain. Standards were prepared by spiking amphetamine in naïve mouse tissue from 0.01–100 µmol/g in a half-log dilution series. Plasma (20 µL) and brain samples and corresponding standards were placed in low retention microfuge tubes with 5 µL/mg of extraction solution for protein precipitation. Samples were vortex mixed, followed by centrifugation at 16,000 × g for 5 min at 4 °C. The supernatants (80 µL) were transferred to a 96 well plate and 2 µL was injected for analysis. Samples were analyzed on an UltiMate 3000 UHPLC coupled to Q Exactive Focus Orbitrap mass spectrometer (Thermo Fisher Scientific). Samples were separated on an Agilent Eclipse Plus C18 RRHD (1.8 µm) 2.1 × 100 mm column. The mobile phase consisted of water + 0.1% formic acid (A), and acetonitrile + 0.1% formic acid (B). Separation was achieved at a flow rate of 0.4 mL/min using a gradient run, from 97.5/2.5 (A/B) to 5/95 (A/B) over 1.5 min, maintaining at 5/95 (A/B) for 1 min, and then re-equilibrating for 1 min. Samples were introduced to the source through heated ion spray with the capillary temperature setting at 350 °C and spray voltage of 3.5 kV. Nitrogen was used as the sheath and auxiliary gas with the settings of 30 and 5 respectively. Quantification was performed in product-reaction monitoring (PRM) mode with a collision energy setting of 10. Transitions 136.1121–119.0876 m/z and 91.0563 m/z (amphetamine) and 423.1695–377.1522 m/z and 207.0915 m/z (losartan) were monitored. Data were acquired and quantified with Xcalibur software.

### Body composition

Body composition was determined by using a nuclear magnetic resonance scanner (EchoMRI-100, Houston, TX).

### Tamoxifen treatment

Tamoxifen (10 mg/ml in corn oil; MilliporeSigma) was administrated daily (100 mg/kg, i.p.) for 6 consecutive days to Tsn ^fl/fl^ and UBC-Cre ^+/−^/Tsn ^fl/fl^ mice. Three weeks later, these mice were used to monitor the locomotor response to amphetamine administration.

### Statistical analysis

Data are presented as mean ± SEM. Statistical significance was evaluated using GraphPad Prism 7 (GraphPad Software, La Jolla, CA). The student’s *t*-test was used to compare groups in single variable experiments. Repeated measures (RM) two-way ANOVA was used to analyze multiple variable experiments. Pairwise comparisons were made using Bonferroni’s post-hoc test. Differences were considered significant at *p* < 0.05.

Sample sizes were based on either pilot studies conducted in our lab or literature reports in which similar paradigms were used.

## Results

### TN and TX are expressed in midbrain DA and striatal neurons

**Fig. 1:**
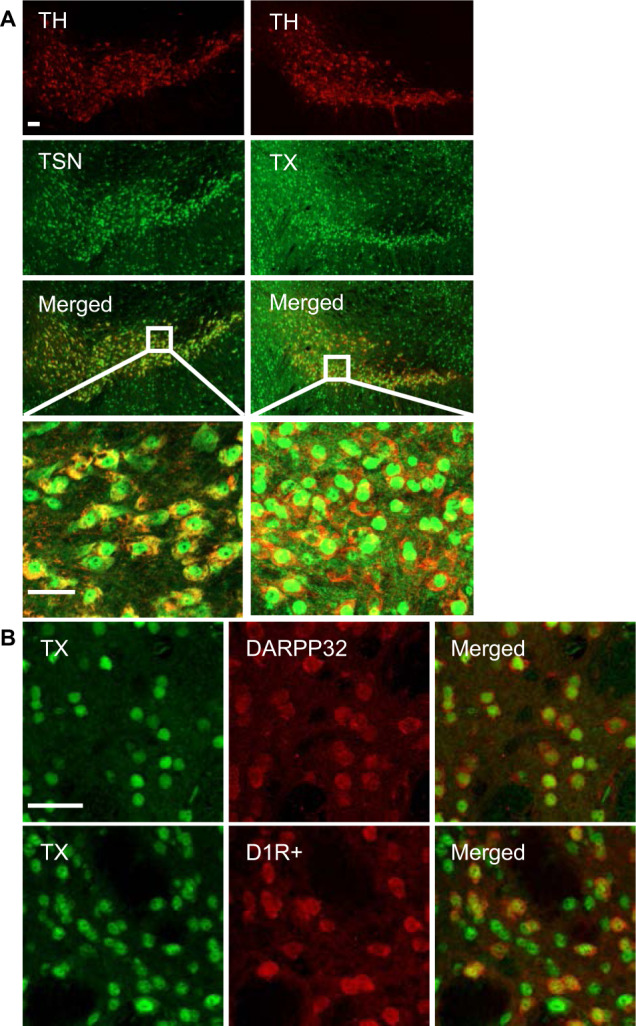
Localization of TN and TX to DA neurons and striatal neurons. **A** Double immunostaining of midbrain sections for TH (red) and TN or TX (green) shows TN or TX expression located in the VTA or substantia nigra. **B** Immunostaining in striatal medium spiny neurons shows prominent nuclear TX staining (green) in DARPP-32 positive neurons (red) (top row). TX staining of sections from mice expressing tdTomato in D1R + neurons demonstrates that TX is expressed in both D1R-positive neurons as well as in D1R-negative neurons (bottom row). Scale bar, 30 μm.

In prior immunostaining studies characterizing the expression of TN and TX in the hippocampus and cortex [7], we noticed prominent neuronal staining in the striatum and several brain stem areas. To check if TN and TX are expressed in midbrain DA neurons, we performed double immunostaining for TH and either TN or TX. These studies demonstrate clear co-expression of TN and TX in DA neurons (Fig. 1A). TX staining in the striatum is present in both D1R-positive and D2R-positive neurons, as we found a high degree of co-expression of TX in neurons stained for DARPP-32, which is expressed in both populations [16]. Furthermore, we conducted TX staining in tdTomato reporter mice (Ai9) that express this marker selectively in D1R-positive neurons, under the control of Drd1-Cre. We found that TX is expressed in both D1R-positive neurons, which are labeled with tdTomato, as well as in presumptive D2R-positive neurons, which are not (Fig. 1B).

### Tsn KO mice display increased locomotor response to amphetamine

**Fig. 2:**
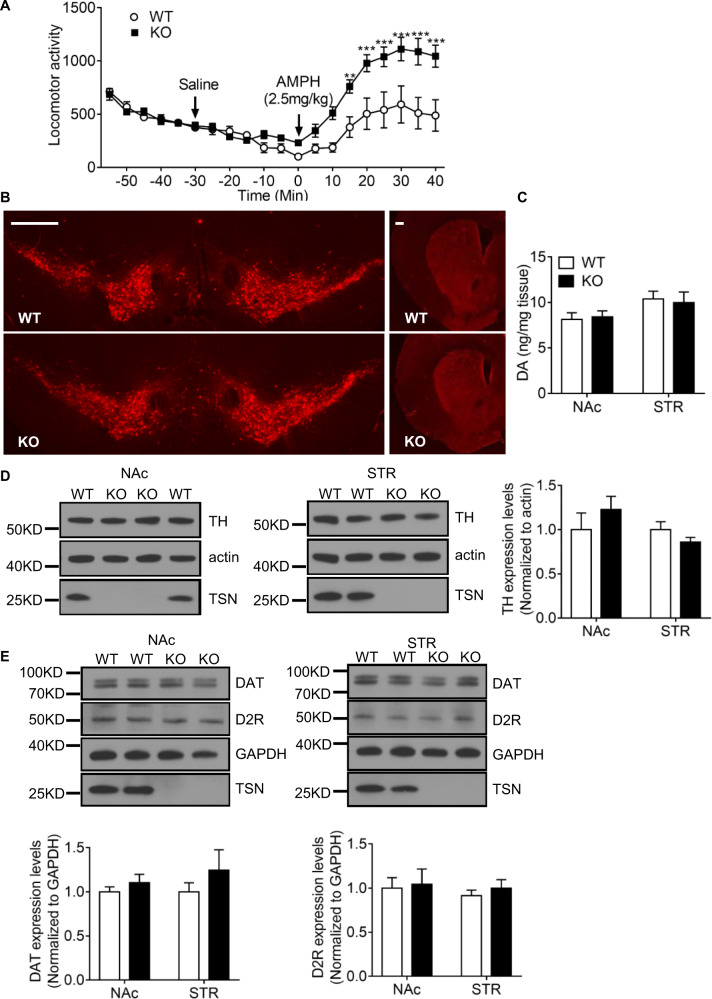
*Tsn* KO mice display increased locomotor response to amphetamine (AMPH) but normal levels of tissue DA, TH, DAT and D2R. **A** Amphetamine-induced (2.5 mg/kg, i.p.) locomotor activity is increased in male KO mice. Locomotor activity was monitored every 5 min and arrowheads indicate the time of injections. *n* = 8/group. Two-way ANOVA with RM revealed a significant effect of time (*p* < 0.0001), a significant effect of genotype (*p* = 0.0095), as well as a significant interaction between time X genotype (*p* < 0.0001). Bonferroni post-hoc testing was done to identify individual time points that were significantly different. **p < 0.01, and ***p < 0.001. **B** Immunostaining shows comparable TH staining of DA neurons (left panel) and striatal terminals (right panel) in WT and translin KO mice. Scale bar, 500 μm. **C** Tissue DA levels in the nucleus accumbens (NAc) and striatum (STR) are similar in WT and translin KO mice. *n* = 5–6/group. **D**, **E** Immunoblotting shows normal TH, DAT, D2R expression levels in NAc and STR in translin KO mice. Image J was used for quantification. *n* = 6/group. Data are expressed as mean ± SEM. Statistical significance was assessed by Student’s *t*-test.

To investigate the role of the microRNA system in regulating behavioral responses to amphetamine, we compared its ability to stimulate open field locomotor activity in *Tsn* KO mice and WT littermates. We found that the locomotor response to amphetamine is markedly enhanced in both male and female *Tsn* KO mice (Fig. 2A and S1). In light of this observation, we checked if *Tsn* deletion impacts the midbrain DA pathway, which plays a major role in mediating the behavioral effects of amphetamine [14, 15].

Qualitatively, staining of the midbrain and striatal sections from WT and *Tsn* KO mice for tyrosine hydroxylase (TH), showed a comparable pattern of expression of this enzyme in dopamine neuronal cell bodies and striatal projections between the two genotypes (Fig. 2B). To obtain a quantitative assessment of the status of dopamine neurons, we measured tissue dopamine levels in nucleus accumbens (NAc) and dorsal striatum (STR) by HPLC (Fig. 2C), as well as protein levels of TH (Fig. 2D), dopamine transporter (DAT) and D2 dopamine receptor (D2R) by immunoblotting (Fig. 2E), in both the NAc and STR. We did not detect any differences between samples obtained from *Tsn* KO mice and WT littermates in these assays.

### Effect of conditional deletion of Tsn or Tsnax on locomotor response to amphetamine

To check whether this phenotype might reflect the loss of the TN/TX RNase from dopaminergic or striatal neurons, we monitored the locomotor response to amphetamine in mice with either conditional deletion of *Tsn* from DA neurons or conditional deletion of *Tsnax* from D2R-positive striatal neurons (Supplementary Text and Figure S2). Neither of these manipulations phenocopied the exaggerated amphetamine response displayed by constitutive *Tsn* KO mice.

**Fig. 3:**
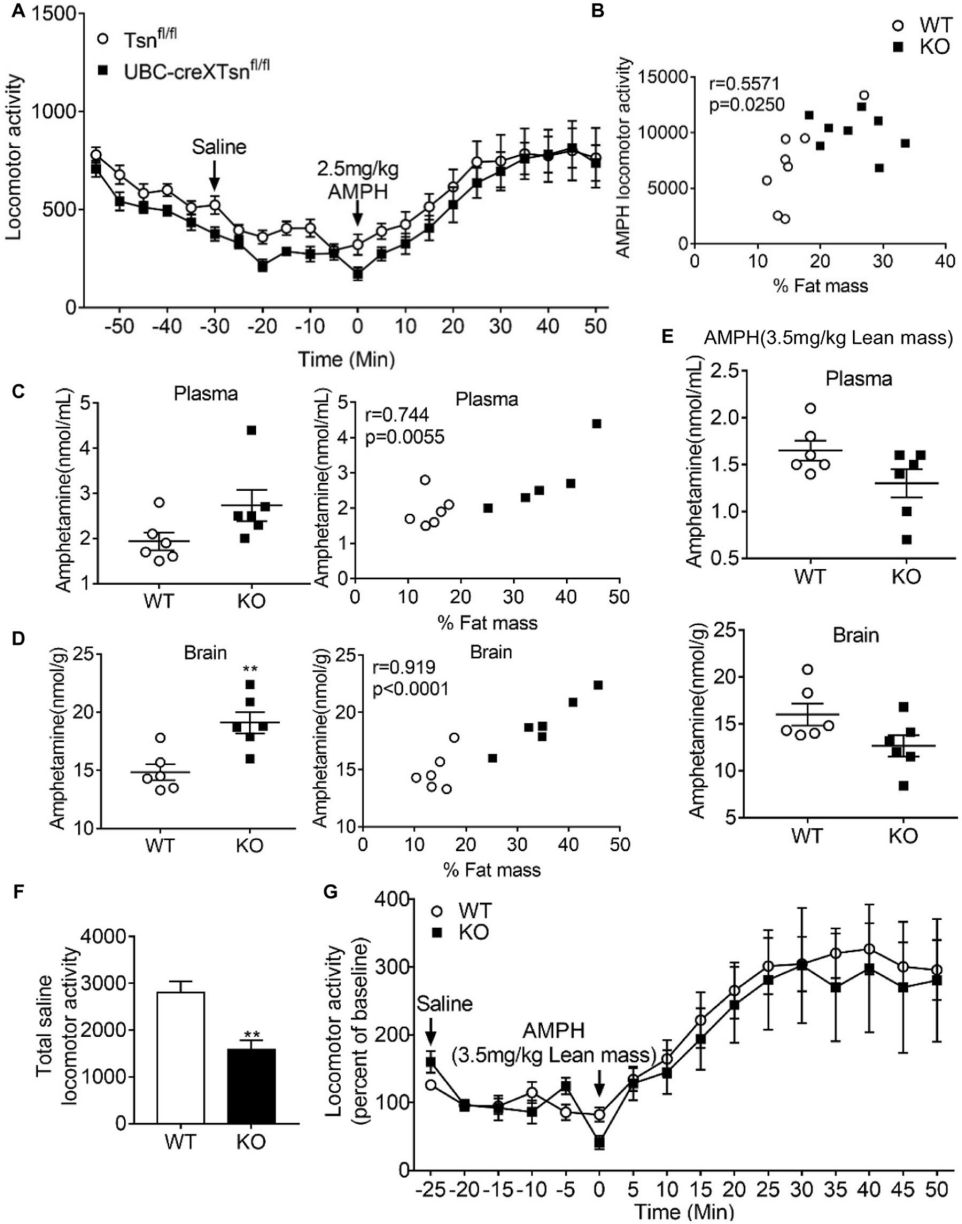
Correlation of fat mass with AMPH-induced locomotor activity and AMPH levels in plasma and brain. **A** Global, conditional deletion of *Tsn* during adulthood does not affect the locomotor response to AMPH (2.5 mg/kg, i.p.). **B** The total locomotor activity induced by AMPH (2.5 mg/kg i.p.) in WT and *Tsn* KO mice was correlated with % fat mass (*r* = 0.5571, *P* = 0.025). *n* = 8/group. **C** Plasma AMPH levels (in nmol/mL) (left), and correlation with % fat mass (right; *r* = 0.744, *P* = 0.0055). 2.5 mg/kg AMPH was injected i.p. based on body weight. *n* = 6/group. **D** Brain AMPH levels (in nmol/g) (left), and correlation with % fat mass (right; *r* = 0.919, *p* < 0.0001). 2.5 mg/kg AMPH was injected i.p. based on body weight. *n* = 6/group. **E** Plasma and brain AMPH levels when 3.5 mg/kg AMPH was injected i.p. based on lean mass. *n* = 6/group. **F** Prior to i.p. injection of 3.5 mg/kg based on lean mass, baseline locomotor activity monitored following saline injection was lower in Tsn KO mice than in WT littermates. **G** The normalized locomotor response to AMPH shown as a percentage of the baseline level (100%) is comparable between groups. *n* = 7-8/group. Data are expressed as Mean ± SEM. Statistical significance indicated by asterisks was assessed by Student’s *t*-test. **p < 0.01.

To assess whether this amphetamine phenotype is due to the absence of the TN/TX RNase in other cell types, we investigated the impact of global, conditional deletion of *Tsn* in adulthood [26]. This manipulation was achieved by tamoxifen treatment of mice that are homozygous for a floxed allele of *Tsn* and hemizygous for the UBC-CreERT2 allele. The response to amphetamine is unchanged in these conditional KO mice (Fig. 3A), indicating that the exaggerated response to amphetamine observed in constitutive *Tsn* KO mice is not due to the absence of the TN/TX RNase at the time of amphetamine administration. Rather, it appears to be secondary to effects induced by *Tsn* deletion during development. In particular, since we have previously demonstrated that this manipulation does not phenocopy the robust adiposity displayed by constitutive *Tsn* KO mice [26], we hypothesized that the exaggerated amphetamine response is due to elevated adiposity, which has been found to impact the pharmacokinetics of many drugs [27–29]. Furthermore, since the amphetamine dose (2.5 mg/kg) used in monitoring locomotor response is in the mid-portion of the ascending part of the dose-response curve [30], increased levels of amphetamine in *Tsn* KO mice could account for their enhanced response in this assay.

### Increased brain levels of amphetamine in Tsn KO mice: correlation with increased adiposity

Consistent with this scenario, we noted a positive correlation between locomotor response to amphetamine and adiposity (%fat mass) that is pronounced in WT mice (Fig. 3B), but also present when including both WT and *Tsn* KO mice. To test this directly, we collected both plasma and forebrain samples at 30 min following administration of amphetamine (2.5 mg/kg, i.p.). As shown in Fig. 3C, we did not detect a significant difference in plasma levels of amphetamine between WT and *Tsn* KO mice. Furthermore, we found only a weak correlation between plasma amphetamine level and %body fat that is likely misleading as it appears to be driven by data from one mouse that displayed the highest amphetamine level and %body fat. In contrast, we found that brain levels of amphetamine are increased in *Tsn* KO mice, with a strong correlation between adiposity and amphetamine levels in the brain (Fig. 3D). Thus, these findings: (1) indicate that the increased locomotor response to amphetamine in *Tsn* KO mice reflects increased brain levels of this drug, and (2) suggest that the alteration in amphetamine pharmacokinetics is related to the elevated adiposity, or other correlated metabolic abnormalities, displayed by *Tsn* KO mice.

Since *Tsn* KO mice exhibit a prominent increase in adiposity (~ 2–3 fold) without an increase in total body weight [12], both WT and *Tsn* KO mice received comparable amounts (2.5 mg/kg of total body weight) of amphetamine in experiments monitoring its effects on locomotor activity. On the other hand, *Tsn* KO mice received a higher dose with respect to their reduced lean body weight. As lipophilic compounds, such as amphetamine, are preferentially distributed to tissues with high blood flow, such as the brain [31], it is conceivable that amphetamine would be preferentially delivered to the brain at the expense of adipose tissue, which has a very low perfusion rate. These findings suggest that calculating the amount of amphetamine administered to each mouse based on their lean body mass, rather than total body weight, should eliminate the disparity in brain amphetamine levels between WT and *Tsn* KO mice. Of note, this is one of the standard approaches recommended for adjusting drug dosage for obese patients [32]. To test this inference, we administered a dose of 3.5 mg/kg amphetamine, i.p., based on lean body weight, and collected plasma and brain samples 30 min later. We selected this dose so that WT mice would receive the same amount of amphetamine with this regimen, based on lean body weight, as they did with the dose of 2.5 mg/kg, based on total body weight. This calculation was based on our finding that WT mice have a lean mass that is on average approximately 74% of their total body weight. Thus, the amounts of drug administered when using a dose of 3.5 mg/kg, based on lean body weight, or 2.5 mg/kg, based on total body weight, are comparable.

Consistent with our hypothesis, this modified dosing regimen produced similar levels of amphetamine in the brain of WT and *Tsn* KO mice (Fig. 3D). Furthermore, as predicted, amphetamine-induced locomotor activity is comparable in WT and Tsn KO mice (Fig. 3G). However, it is important to keep two caveats in mind in interpreting these results. First, by basing the dosing regimen on lean body weight rather than total body weight, the Tsn KO mice receive less amphetamine, which would be expected to decrease their brain amphetamine levels. Second, this cohort of *Tsn* KO mice displayed lower baseline activity prior to administration of amphetamine (Fig. 3F). Therefore, it is possible to interpret the locomotor response to amphetamine in two ways. If, as shown in Fig. 3G, we plot the post-amphetamine locomotor activity as a percentage of baseline activity, the WT and *Tsn* KO mice show comparable responses. On the other hand, if one considers the absolute increase in locomotor response induced by amphetamine relative to baseline, then *Tsn* KO mice would show a decreased response compared to WT mice. However, our observation that conditional deletion of *Tsn* in adult mice does not affect baseline level of activity or the locomotor response to amphetamine (Fig. 3A) supports the view that *Tsn* deletion does not impair the neuronal action of amphetamine that mediates its classic locomotor stimulatory effect.

### Increased adiposity in WT mice elevates brain amphetamine levels

Although these findings indicate that the robust adiposity present in *Tsn* KO mice elicits elevated brain amphetamine levels, they do not rule out the possibility that the elevated brain amphetamine levels detected in *Tsn* KO mice could be due to other effects of *Tsn* deletion that are correlated with their elevated adiposity. Accordingly, to exclude this potential confound, we assessed the effects of diet-induced adiposity on amphetamine brain levels in WT mice. For this experiment, we used the initial dosing regimen of 2.5 mg/kg based on total body weight because we wanted to check whether amphetamine brain levels and locomotor response are also elevated in WT mice with elevated adiposity, as found in *Tsn* KO mice.

**Fig. 4:**
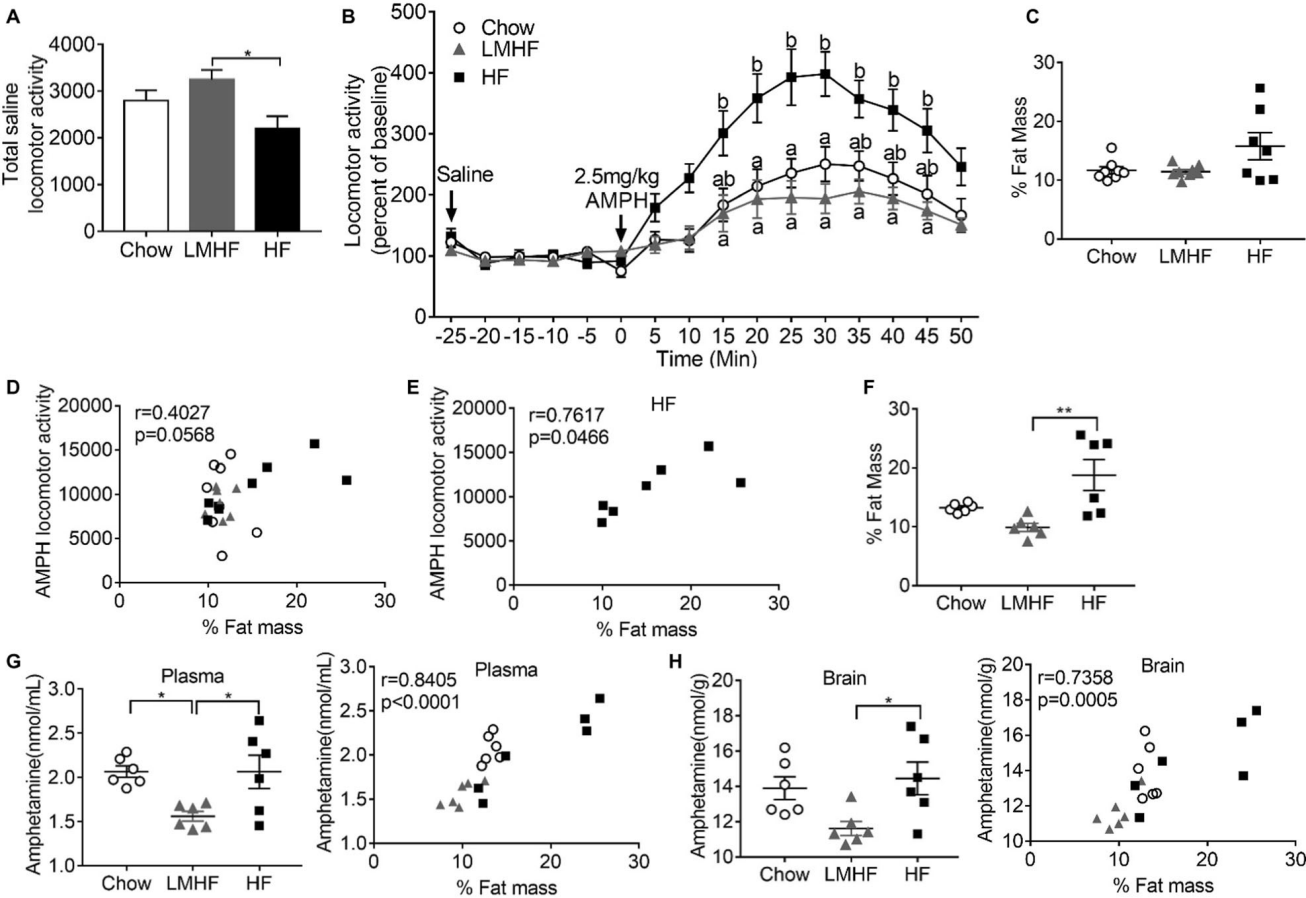
Effect of HF diet and adiposity on AMPH-induced locomotor activity and AMPH levels in plasma and brain. **A** The baseline locomotor activity was lower in WT mice fed the HF diet (HF), compared to those that were given limited access to HF diet (LMHF). *n* = 8/group. **B** The normalized locomotor response to AMPH is higher in HF mice compared to Chow or LMHF mice. Two-way ANOVA with RM revealed a significant effect of time (*p* < 0.0001), a significant effect of group (*p* = 0.0015), as well as a significant interaction between the time X group (*p* = 0.0001). Different letters indicate statistically significant differences (*p* < 0.05) at individual time points based on Bonferroni post-hoc testing. **C** Adiposity (% Fat Mass) values for each mouse used for locomotor testing. **D** Correlation between % fat mass and AMPH locomotor activity for mice in all three groups falls just outside the significance threshold (*p* = 0.0568). **E** Limiting correlation to HF mice yields a *p* value (0.0466) just within the threshold for significance. **F** Adiposity (% Fat Mass) values for each mouse used for determination of amphetamine levels are shown. Adiposity of the HF group is significantly greater than that of the LMHF group. *n* = 6/group. **G** Panel at left shows that plasma amphetamine levels are higher in Chow or HF groups compared to LMHF group. The right panel shows a strong correlation between % fat mass and plasma amphetamine levels (*p* < 0.0001). **H** Panel at left shows that brain amphetamine levels in the HF group are higher than those in the LMHF group. The panel at right shows a strong correlation between % fat mass and brain amphetamine levels (*p* = 0.0005).

WT mice were separated into three groups: one group was maintained on regular chow (Chow), one group was switched to an HF diet (HF), and the third group had limited access to the HF diet (LMHF; 2 h on MWF). After seven weeks on these regimens, mice were scanned to determine their %body fat, and then used to assay locomotor response to amphetamine or measure amphetamine levels in plasma and brain. Comparison of the baseline activity levels following administration of saline revealed that mice in the HF group had reduced activity compared to the LMHF mice (Fig. 4A). Analysis of locomotor activity following amphetamine administration demonstrated that the HF mice exhibited an elevated response to amphetamine compared to the other two groups (Fig. 4B).

Examination of the %body fat across these groups revealed that the HF group shows a wide range of values (Fig. 4C), with three mice displaying elevated adiposity and the other three mice having %body fat in the same range as mice in the other groups. This spread in %body fat among mice in the HF group is expected [33] and allowed us to assess if there is a correlation between adiposity and amphetamine-induced locomotor activity. Correlation analysis of mice across all three groups indicates a trend that is just outside the significant range (Fig. 4D). Restricting the correlation analysis to the HF group yields a correlation that is just within the significant range. However, analysis of the effect of adiposity on amphetamine levels in plasma and the brain revealed a strong correlation between these variables (Fig. 4F–H). Taken together, these findings indicate that elevated adiposity elicits increased brain amphetamine levels which would account for the increased locomotor response to amphetamine displayed by HF mice and *Tsn* KO mice.

## Discussion

The major finding of this study is that elevated adiposity increases brain amphetamine levels. This inference is based on evidence obtained from both a genetic model of increased adiposity, constitutive *Tsn* KO mice, and diet-induced adiposity in WT mice. Previous studies analyzing the heightened locomotor response to amphetamine of *ob/ob* mice, which display extreme obesity, revealed that these mice have higher brain levels of amphetamine [34]. However, it is not clear from that classical study of *ob/ob* mice or our current findings in *Tsn* KO mice whether the increase in brain amphetamine levels is due to the increased adiposity or another effect of the genetic manipulation. Accordingly, it was essential to check whether increased adiposity induced in WT mice by consumption of an HF diet also leads to increased levels of brain amphetamine.

Our working model posits that the elevated brain levels of amphetamine observed in *Tsn* KO mice are due to their robust adiposity in the context of normal body weight. As a result, these mice received a higher dose per lean body mass than WT mice, which could yield higher brain levels. Consistent with this view, we confirmed that calculating the dose of amphetamine-based on lean body mass corrects the disparity in brain amphetamine levels. However, since these studies were performed with *Tsn* KO mice, they left open the possibility that the elevated brain levels of amphetamine might be secondary to other effects caused by constitutive deletion of this gene rather than increased adiposity. Therefore, to exclude this possibility we confirmed that elevated adiposity induced by switching WT mice to an HF diet also elevates brain levels of amphetamine.

As HF feeding has been shown to increase blood-brain barrier permeability [35–37], it is conceivable that this mechanism could account for increased brain amphetamine levels. However, since amphetamine, a lipophilic compound, gains access to the brain readily in normal mice, it is not clear that this mechanism mediates the higher brain amphetamine levels observed in this study. Another attractive mechanism posits that the elevated brain levels of amphetamine reflect the preferential distribution of lipophilic compounds, such as amphetamine, to tissues with high blood flow, such as the brain [31]. As adipose tissue has a very low perfusion rate, but accounts for an abnormally large percentage of body mass in *Tsn* KO mice and mice with HF-diet induced obesity, compared to control mice, dosing of animals based on their total body weight would, according to this model, lead to elevated brain levels of amphetamine.

Amphetamine and its analogues are widely used to treat attention deficit hyperactivity disorder (ADHD) [38, 39]. Our unexpected observation that adiposity impacts brain amphetamine levels suggest that further investigations are warranted to assess this correlation in human subjects. Furthermore, this issue is of particular concern since it is well established that ADHD is associated with obesity [40], raising the prospect that the use of standard dosing regimens in obese patients produces higher levels of amphetamine in the brain. In addition, it may be important to check if the therapeutic response or side effects produced by amphetamine may correlate with adiposity. If our findings extend to patients, then taking adiposity data into account may help determine the amphetamine dosing regimen that will optimize therapeutic efficacy.

## Supplementary information

Supplementary Text

Figure S1

Figure S2
