# Supplementary material for: Elevated body fat increases amphetamine accumulation in brain: evidence from genetic and diet-induced forms of adiposity
Source: Transl Psychiatry. 2021 Aug 14;11:427. doi: 10.1038/s41398-021-01547-9 (PMC8364554; doi:10.1038/s41398-021-01547-9)
Supplement: Supplementary file 1 — Supplementary Text [file 41398_2021_1547_MOESM1_ESM.docx]

**Supplementary Text**

As midbrain DA neurons express TN and TX and are the primary site of action of amphetamine, we proceeded to check whether selective deletion of *Tsn* from DA neurons or *Tsnax* from D2R-positive striatal neurons phenocopy the exaggerated locomotor response to amphetamine displayed by constitutive *Tsn* KO mice. To assess this possibility, we generated mice that are homozygous for the floxed *Tsn* allele, *Tsn* ^fl/fl^, and hemizygous for the DAT-Cre allele (1). Double immunostaining for TN and TH demonstrated complete loss of TN staining in midbrain DA neurons (Figure S2). Furthermore, as found in *Tsn* KO mice (2), conditional deletion of *Tsn* from DA neurons also induces loss of TX protein. We then evaluated the impact of selective deletion of *Tsn* from DA neurons on the locomotor response to amphetamine. Since these mice are both hemizygous for the DAT-Cre allele and homozygous for the *Tsn* floxed allele, we also tested control mice that have each of these genotypic changes separately. Comparison of the effect of amphetamine on locomotor activity in these three groups indicated that conditional deletion of *Tsn* from DA neurons does not increase the locomotor response to amphetamine above that displayed by mice that are hemizygous for the DAT-Cre allele. However, these results need to be interpreted cautiously, since, consistent with the recent report by Chohan et al. (3), control mice that are hemizygous for the DAT-Cre allele exhibit a reduced response to amphetamine compared to that of the other control group, mice that are homozygous for the *Tsn* floxed allele.

Previous studies have demonstrated that deletion of D2 receptors from D2R-positive striatal neurons blocks the locomotor response to methamphetamine (4,5). Furthermore, TX binds to the C-terminal tail of the A2a adenosine receptor, which is selectively expressed in D2R-positive striatal neurons (6). Accordingly, we used A2a adenosine receptor-Cre mice (Adora2a-Cre KG139) to check whether conditional deletion of *Tsnax*, the gene encoding TX, from D2R-positive striatal neurons affects the locomotor response to amphetamine. We confirmed that these mice lack TX staining in about half of striatal neurons, as expected (Figure 5C). The locomotor response to amphetamine in these mice was identical to that observed in Tsnax ^fl/fl^ mice demonstrating that the increased response to amphetamine observed in constitutive Tsn KO mice is not mediated by loss of the TN/TX complex from D2R-positive striatal neurons (Figure S2).

**References**

1.Backman, C.M., N. Malik, Y. Zhang, L. Shan, A. Grinberg, B.J. Hoffer, et al., Characterization of a mouse strain expressing Cre recombinase from the 3' untranslated region of the dopamine transporter locus. Genesis, 2006. 44(8): p. 383-90.

2. Chennathukuzhi, V., J.M. Stein, T. Abel, S. Donlon, S. Yang, J.P. Miller, et al., Mice deficient for testis-brain RNA-binding protein exhibit a coordinate loss of TRAX, reduced fertility, altered gene expression in the brain, and behavioral changes. Mol Cell Biol, 2003. 23(18): p. 6419-34.

3. Chohan, M.O., S. Esses, J. Haft, S. Ahmari, and J. Veenstra-VanderWeele, Altered baseline and amphetamine-mediated behavioral profiles in dopamine transporter Cre (DAT-Ires-Cre) mice compared to tyrosine hydroxylase Cre (TH-Cre) mice. Psychopharmacology (Berl), 2020.

4. Kelly, M.A., M.J. Low, M. Rubinstein, and T.J. Phillips, Role of dopamine D1-like receptors in methamphetamine locomotor responses of D2 receptor knockout mice. Genes Brain Behav, 2008. 7(5): p. 568-77.

5. Neve, K.A., C.P. Ford, D.C. Buck, D.K. Grandy, R.L. Neve, and T.J. Phillips, Normalizing dopamine D2 receptor-mediated responses in D2 null mutant mice by virus-mediated receptor restoration: comparing D2L and D2S. Neuroscience, 2013. 248: p. 479-87.

6. Chien, T., Y.T. Weng, S.Y. Chang, H.L. Lai, F.L. Chiu, H.C. Kuo, et al., GSK3beta negatively regulates TRAX, a scaffold protein implicated in mental disorders, for NHEJ-mediated DNA repair in neurons. Mol Psychiatry, 2018. 23(12): p. 2375-2390.

**Supplementary Figure Legends**

**Figure S1: Elevated locomotor response to amphetamine in female *Tsn* KO mice.**

Amphetamine-induced (2.5 mg/kg, i.p.) locomotor activity is increased in female KO mice. Locomotor activity was monitored every 5 minutes and arrow indicate time of injections. n = 8/group. Two-way ANOVA with RM revealed a significant effect of time (p<0.0001), as well as a significant interaction between time X genotype (p<0.0001). Bonferroni post-hoc testing was done to identify individual time points that were significantly different. **p<0.01, and ***p<0.001.

**Figure S2: Conditional deletion of *Tsn* from DA neurons or of *Tsnax* from D2R-positive neurons.**

(A) Double immunostaining of midbrain sections for TH (red) and TN or TX (green) shows that TN or TX expression was totally absent in DAT-Cre xTsn^fl/fl^ mice. Scale bar, 50 μm. (B) AMPH-induced (2.5 mg/kg i.p.) locomotor activity in DAT-Cre xTsn^fl/fl^ mice was not significantly different from the response of mice that are hemizygous for the DAT-Cre allele. n=8/group. Data are expressed as mean ± SEM. Different letters indicate statistically significant differences (p < 0.05) by two-way ANOVA with repeated measures followed by Bonferroni’s post-hoc test. (C) Immunostaining of striatal sections for TX (green) shows TX expression is absent from about half of cells in A2a-Cre xTsnax^fl/fl^ mice. Red arrows point to striatal neurons that lack TX staining. (D) AMPH-induced (2.5 mg/kg i.p.) locomotor activity is unaffected in A2a-Cre xTsnax^fl/fl^ mice compared to Tsnax^fl/fl^ mice. n =8/group. Data are expressed as mean ± SEM. Statistical significance was assessed by repeated measure two-way ANOVA followed by Bonferroni’s post-hoc analysis.
