# Supplementary figures and images for: Elevated body fat increases amphetamine accumulation in brain: evidence from genetic and diet-induced forms of adiposity
Source: Transl Psychiatry. 2021 Aug 14;11:427. doi: 10.1038/s41398-021-01547-9 (PMC8364554; doi:10.1038/s41398-021-01547-9)

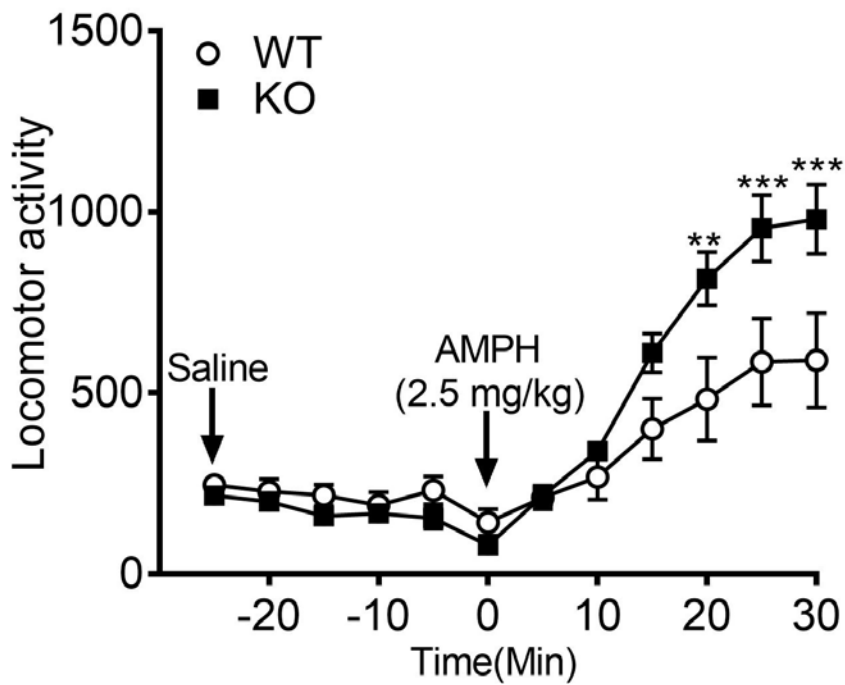

Supplement: Supplementary file 2 — Figure S1 [file 41398_2021_1547_MOESM2_ESM.pdf]
