# Supplementary material for: Elevated body fat increases amphetamine accumulation in brain: evidence from genetic and diet-induced forms of adiposity
Source: Transl Psychiatry. 2021 Aug 14;11:427. doi: 10.1038/s41398-021-01547-9 (PMC8364554; doi:10.1038/s41398-021-01547-9)
Supplement: Supplementary file 3 — Figure S2 [file 41398_2021_1547_MOESM3_ESM.pdf]

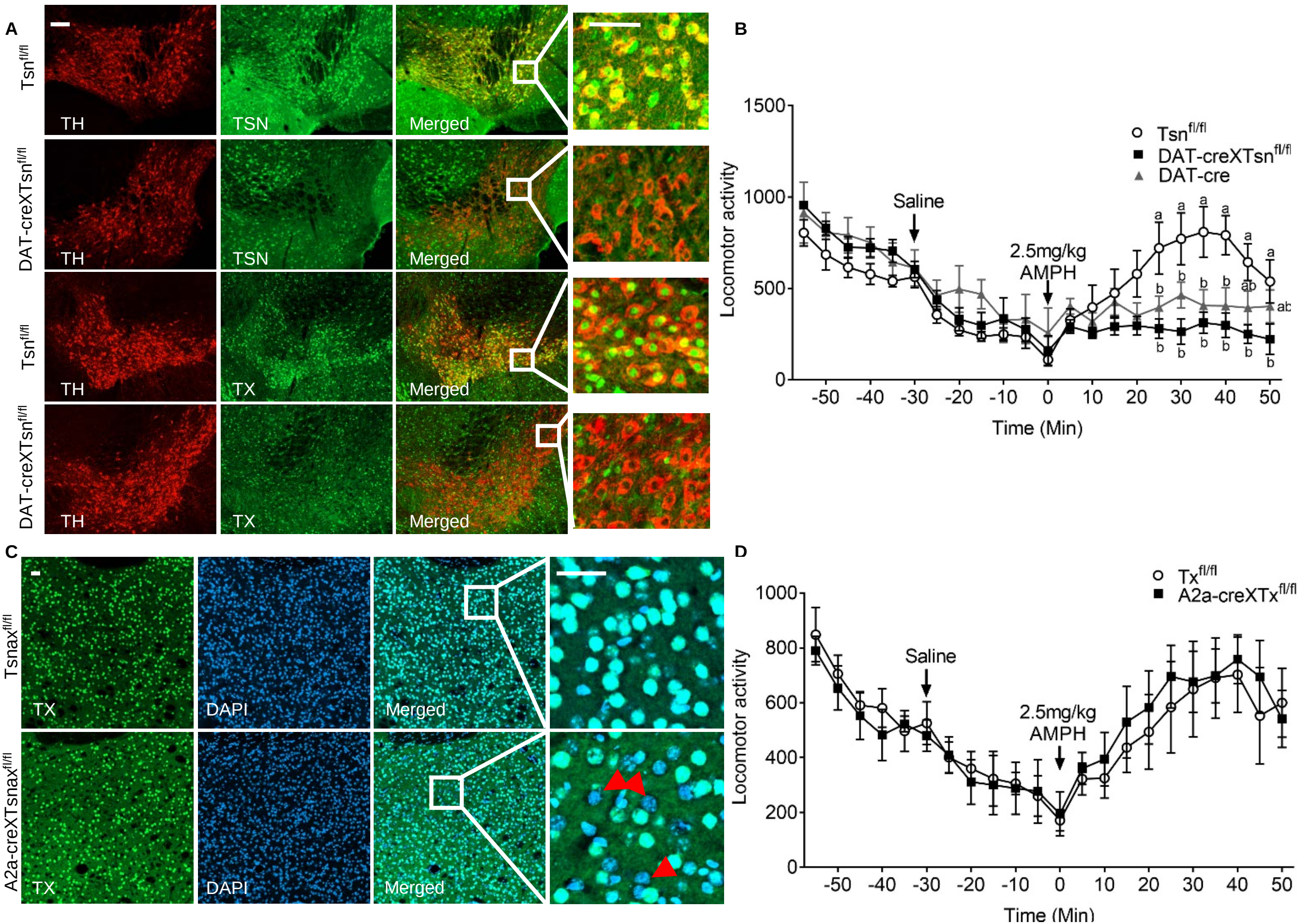

A
Tsn^fl/fl
DAT-creXTsn^fl/fl
TH
TSN
TX
Merged
B
Tsn^fl/fl
DAT-creXTsn^fl/fl
DAT-cre
Saline
2.5mg/kg AMPH
Locomotor activity
Time (Min)
C
Tsnax^fl/fl
A2a-creXTsnax^fl/fl
TX
DAPI
Merged
D
Tx^fl/fl
A2a-creXTx^fl/fl
Saline
2.5mg/kg AMPH
Locomotor activity
Time (Min)
